# Size-Based Isolation of Circulating Tumor Cells in Lung Cancer Patients Using a Microcavity Array System

**DOI:** 10.1371/journal.pone.0067466

**Published:** 2013-06-28

**Authors:** Masahito Hosokawa, Hirotsugu Kenmotsu, Yasuhiro Koh, Tomoko Yoshino, Takayuki Yoshikawa, Tateaki Naito, Toshiaki Takahashi, Haruyasu Murakami, Yukiko Nakamura, Asuka Tsuya, Takehito Shukuya, Akira Ono, Hiroaki Akamatsu, Reiko Watanabe, Sachiyo Ono, Keita Mori, Hisashige Kanbara, Ken Yamaguchi, Tsuyoshi Tanaka, Tadashi Matsunaga, Nobuyuki Yamamoto

**Affiliations:** 1 Division of Biotechnology and Life Science, Institute of Engineering, Tokyo University of Agriculture and Technology, Tokyo, Japan; 2 Drug Discovery and Development Division, Shizuoka Cancer Center Research Institute, Shizuoka, Japan; 3 Division of Thoracic Oncology, Shizuoka Cancer Center, Shizuoka, Japan; 4 Division of Diagnostic Pathology, Shizuoka Cancer Center, Shizuoka, Japan; 5 Clinical Trial Coordination Office, Shizuoka Cancer Center, Shizuoka, Japan; 6 Hitachi Chemical Co., Ltd., Tokyo, Japan; Queen Elizabeth Hospital, Hong Kong

## Abstract

**Background:**

Epithelial cell adhesion molecule (EpCAM)-based enumeration of circulating tumor cells (CTC) has prognostic value in patients with solid tumors, such as advanced breast, colon, and prostate cancer. However, poor sensitivity has been reported for non-small cell lung cancer (NSCLC). To address this problem, we developed a microcavity array (MCA) system integrated with a miniaturized device for CTC isolation without relying on EpCAM expression. Here, we report the results of a clinical study on CTCs of advanced lung cancer patients in which we compared the MCA system with the CellSearch system, which employs the conventional EpCAM-based method.

**Methods:**

Paired peripheral blood samples were collected from 43 metastatic lung cancer patients to enumerate CTCs using the CellSearch system according to the manufacturer’s protocol and the MCA system by immunolabeling and cytomorphological analysis. The presence of CTCs was assessed blindly and independently by both systems.

**Results:**

CTCs were detected in 17 of 22 NSCLC patients using the MCA system versus 7 of 22 patients using the CellSearch system. On the other hand, CTCs were detected in 20 of 21 small cell lung cancer (SCLC) patients using the MCA system versus 12 of 21 patients using the CellSearch system. Significantly more CTCs in NSCLC patients were detected by the MCA system (median 13, range 0–291 cells/7.5 mL) than by the CellSearch system (median 0, range 0–37 cells/7.5 ml) demonstrating statistical superiority (p = 0.0015). Statistical significance was not reached in SCLC though the trend favoring the MCA system over the CellSearch system was observed (p = 0.2888). The MCA system also isolated CTC clusters from patients who had been identified as CTC negative using the CellSearch system.

**Conclusions:**

The MCA system has a potential to isolate significantly more CTCs and CTC clusters in advanced lung cancer patients compared to the CellSearch system.

## Introduction

Lung cancer is the leading cause of cancer-related death in most industrialized countries. Small cell lung cancer (SCLC) accounts for approximately 15% of lung cancer cases, and non-small cell lung cancer (NSCLC), which includes adenocarcinoma (ADC) and squamous cell carcinoma (SCC), accounts for 85% of lung cancer cases. It has recently been shown that identification of NSCLC patients by detection of genetic aberrations, specifically *EGFR*-activating mutations and the *EML4-ALK* fusion gene, allows for better prediction of response to EGFR tyrosine kinase inhibitors and ALK inhibitors, respectively [1], [2]. Despite advances in prevention and treatment, NSCLC patients are often diagnosed at an advanced stage and have a poor prognosis due to the disease’s tendency toward distant metastasis, the primary cause of mortality among NSCLC patients. Characterized by aggressive tumor growth and often presenting with metastases in the regional nodes and distant organs, SCLC is initially highly sensitive to chemotherapy but tends to acquire chemoresistance, leading to inevitable relapse.

Circulating tumor cells (CTCs) are defined as tumor cells circulating in the peripheral blood of patients with metastatic cancer. When measured using the US Food and Drug Administration (FDA)-approved CellSearch system (Veridex, Raritan, NJ, USA), the number of CTCs in peripheral blood can be used to predict the prognosis of patients with metastatic breast cancer [3], colorectal cancer [4], prostate cancer [5], NSCLC [6], and SCLC [7]. The CellSearch system enriches CTCs using magnetic beads coated with a monoclonal antibody-targeting epithelial cell marker, such as the epithelial cell-adhesion molecule (EpCAM) [8], [9]. However, several studies have shown that the presence of EpCAM on tumor cells varies with tumor type [10], [11]. The expression of epithelial cell markers, including EpCAM, is downregulated to increase invasiveness and metastatic potential by epithelial-to-mesenchymal transition (EMT) [12]–[16]. It has been suggested that the low prevalence of CTCs detected in patients with advanced NSCLC using the CellSearch system may be due to the loss of EpCAM expression [17], indicating that EpCAM-based CTC isolation methods cannot achieve stable and reproducible CTC recovery from all tumor types.

Other CTC isolation methods are mainly based on differences in the size and deformability between CTCs and hematologic cells. As tumor cells (>8 µm) are larger than leukocytes [18]–[21], isolation by size of epithelial tumor cells (ISET) can be achieved using filtration to separate individual cells. ISET using a polycarbonate filter, an inexpensive, user-friendly method of enriching CTCs, enables the recovery and detection of epithelial-marker-negative CTCs on the basis of size-dependent CTC isolation. In clinical tests, use of an ISET-based system has been found to achieve higher CTC detection sensitivity in patients with metastatic lung cancer compared to use of the CellSearch system [22]–[24].

Recently, microfabricated devices for size-based separation of tumor cells have been widely developed to enable precise and efficient enrichment of CTCs from whole blood [25]–[28]. These devices include a miniaturized microcavity array (MCA) system that we developed for the highly efficient entrapment of single cells by filtration based on differences in the sizes of cells [29], [30]. In a previous study, we examined the application of our MCA system to the detection of spiked tumor cells from unprocessed human whole blood based on differences in the size and deformability between tumor cells and other blood cells [31]. Using our device, we were able to entrap tumor cells onto size- and geometry-controlled microcavity arrays composed of 10,000 apertures by applying negative pressure, allowing the entrapped cells to be easily enumerated and analyzed by microscopic imaging of specified areas. Furthermore, we found that use of the miniaturized device allowed for introduction of a series of reagents for detection of tumor cells through the microfluidic structure. Our results indicate that our system is a simple yet precise system for the detection of tumor cells within whole blood. To confirm and build on our previous findings, we compared the capacity and efficiency of our novel MCA system and the current gold standard CellSearch system in performing CTC detection and enumeration in whole blood samples drawn from a cohort of NSCLC and SCLC patients.

## Materials and Methods

### Study Design and Ethics Statement

This prospective study was conducted to evaluate CTC enumeration using the CellSearch system and the MCA system in patients with metastatic lung cancer in a blinded experiment (UMIN clinical trial registry, number UMIN000005189). The presence of CTCs was assessed individually according to their criteria before knowing any results from each other. The study inclusion criteria were diagnosis of pathologically proven lung cancer with radiologically evident metastatic lesions, i.e., histologically or cytologically confirmed metastatic NSCLC or SCLC, and enrollment at the Shizuoka Cancer Center. The institutional review boards of the Shizuoka Cancer Center approved the study protocol, and all patients provided written informed consent. From each of the 43 patients who were enrolled, among whom 22 had been diagnosed with NSCLC and 21 with SCLC, 10–15 mL of blood was collected in EDTA tubes for CTC enumeration by the MCA system in our laboratory (Shizuoka Cancer Center, Shizuoka, Japan) and 20 mL was collected in CellSave collection tubes for CTC enumeration by the CellSearch system in the laboratory of SRL Inc. (Tokyo, Japan).

### Cell Culture and Labeling

HCC827, NCI-H358, NCI-H441, DMS79, NCI-H69, and NCI-H82 cell lines were purchased from the American Type Culture Collection without further testing or authentication. A549 (Riken Bioresource Center, Tsukuba, Japan) and PC-14 [32] were kindly provided by Dr. Fumiaki Koizumi (National Cancer Center, Tokyo, Japan). The A549, HCC827, NCI-H358, NCI-H441, PC-14, DMS79, NCI-H69, and NCI-H82 NSCLC and SCLC cell lines were cultured in RPMI 1640 medium containing 2 mM of l-glutamine (Sigma-Aldrich, Irvine, UK), 10% (v/v) fetal bovine serum (FBS; Invitrogen Corp., Carlsbad, CA, USA), and 1% (v/v) penicillin/streptomycin (Invitrogen Corp.) for 3–4 days at 37°C with 5% CO_2_ supplementation. Immediately prior to each experiment, cells grown to confluence were trypsinized and resuspended in phosphate-buffered saline (PBS). As a measurement of tumor cell size, cell size distribution was determined using the CASY® Cell Counter+Analyzer System Model TTC (Schärfe System GmbH, Reutlingen, Germany). To evaluate device performance, the tumor cell lines were labeled with CellTracker Red CMTPX (Molecular Probes, Eugene, OR, USA), with labeling achieved by incubating the cells with a tracking dye (5 µM) for 30 min. After the cells had been pelleted by centrifugation (200 g for 5 min), the supernatant was decanted. The cells were then washed twice with PBS to remove any excess dye before being resuspended in PBS containing 2 mM EDTA and 0.5% bovine serum albumin (BSA).

### Fabrication of the MCA System

**Figure 1:**
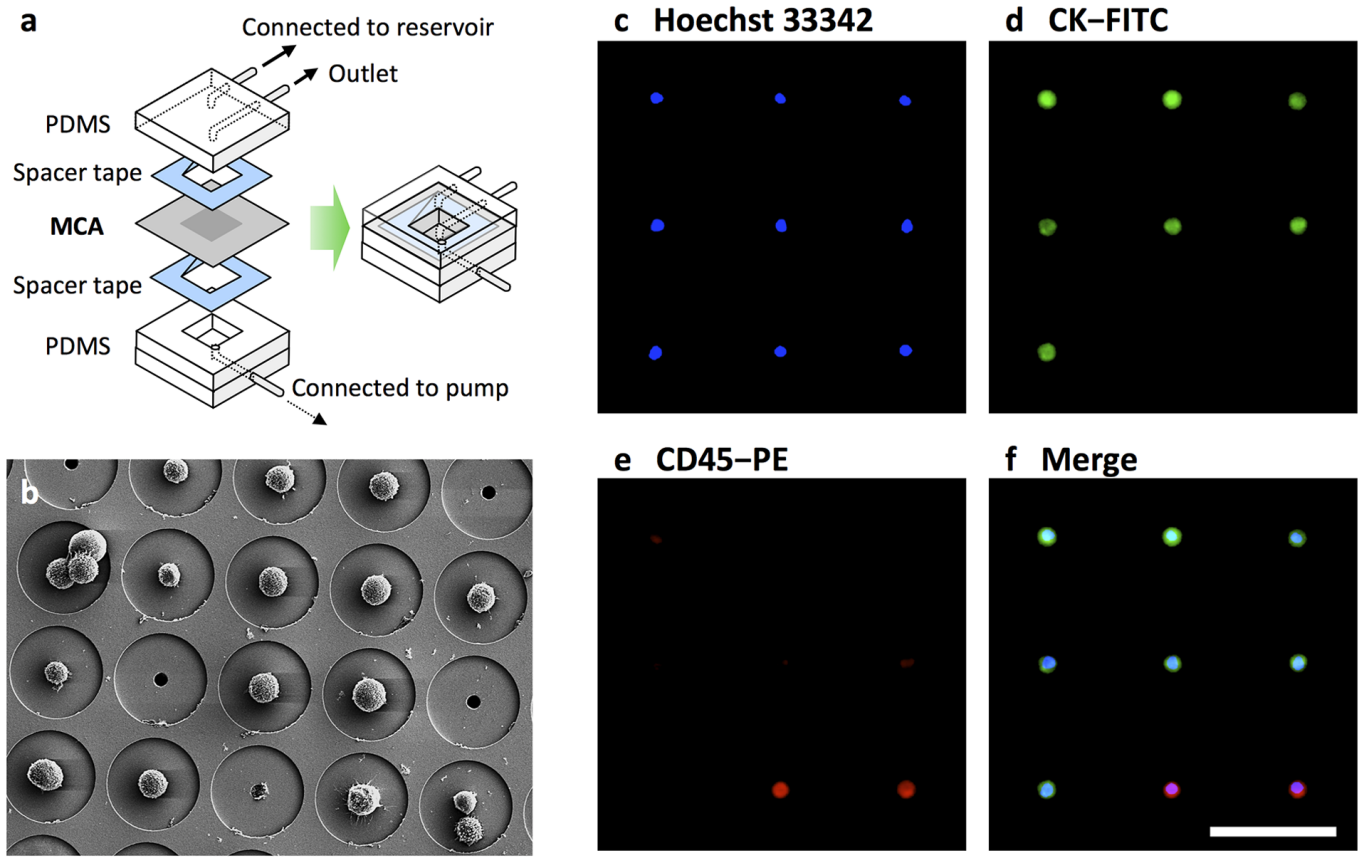
MCA system for size-based isolation of CTCs. (a) Schematic diagram of the structure of the MCA system. (b) Scanning electron microscope image of a cultured tumor cell line trapped on the MCA system. (c–f) Cells isolated from SCLC patient blood stained with Hoechst 33342 (c) and fluorescent-labeled antibodies that target cytokeratin (d) and CD45 (e). Merging of the images (f) allowed for identification of CTCs and hematologic cells. Scale bar = 60 µm.

The MCA system was fabricated in the same manner as previously reported [29], [31]. For CTC enumeration with fluorescence microscope observation, an MCA that had been manufactured by electroforming of nickel was used. For CTC morphological analysis by Giemsa staining, a transparent MCA that had been manufactured by laser irradiation of poly(ethylene terephthalate) (PET) was used. Each of the 10,000 cavities arranged in each 100×100 array was fabricated to have a diameter of 8–9 µm at the top surface and to be 60 µm distant from the adjacent microcavity. Poly(dimethylsiloxane) (PDMS) structures were fabricated and then integrated with the MCA such that the upper substrate consisted of a microchamber, a sample inlet, and an outlet, while the lower substrate beneath the MCA contained a vacuum line to produce negative pressure, enabling cell entrapment. The CTC isolation device was constructed by assembling the MCA, while the upper and lower PDMS layers were constructed using spacer tapes (Figure 1a). The sample inlet was connected to a reservoir, while the vacuum microchannel was connected to a peristaltic pump.

### CTC Enumeration using the MCA System

Human blood samples were collected in a collection tube with EDTA to prevent coagulation and used within 2 h. The average volume of blood analyzed was 4.0 mL per sample (range, 3.0–7.5 mL). All CTC enumeration using the MCA system was performed without knowledge of patient clinical status in the laboratory of the Shizuoka Cancer Center Research Institute. After introduction of blood samples into the reservoir, negative pressure was applied to a cell suspension using a peristaltic pump connected to a vacuum line, allowing the sample to be passed through the microcavities at a flow rate of 200 µL/min. To remove any blood cells remaining on the array, PBS containing 2 mM EDTA and 0.5% BSA (1 mL) was introduced into the reservoir and passed through the microcavities at a flow rate of 200 µL/min for 5 min.

To stain the CTCs with anti-pancytokeratin antibody, trapped cells were fixed by flowing 400 µL of 1% paraformaldehyde (PFA) in PBS through the MCA at a flow rate of 20 µL/min for 20 min. After washing with 100 µL of PBS, the cells were treated with 300 µL of 0.2% Triton X-100 in PBS at a flow rate of 20 µL/min for 15 min. After permeabilization, cells were treated with 3% BSA in PBS at a flow rate of 20 µL/min for 30 min. To identify CTCs and leukocytes, 600 µL of cell-staining solution containing 1 µg/mL of Hoechst 33342 (Molecular Probes); a cocktail of anti-pancytokeratin antibodies (Alexa488-AE1/AE3 (1∶100 dilution; eBioscience, San Diego, CA, USA) and FITC-CK3-6H5 (1∶60 dilution; Miltenyi Biotec, Auburn, California CA USA); and PE-labeled anti-CD45 antibody (1∶120 dilution; BD Biosciences, San Jose, CA, USA) was flowed through the microcavities at a flow rate of 20 µL/min for 30 min. Finally, the array was washed with 400 µL of PBS containing 2 mM of EDTA and 0.5% BSA to remove any excess dye. After recovery of tumor cells, an image of the entire cell array area was obtained using a fluorescence microscope (BX61; Olympus Corporation, Tokyo, Japan) integrated with a 10× objective lens and a computer-operated motorized stage; WU, NIBA, and WIG filter sets; a cooled digital camera (DP-70; Olympus Corporation); and Lumina Vision acquisition software (Mitani Corporation, Tokyo, Japan).

In clinical trials, an entire image of the cell array area had been obtained using a fluorescence microscope (Axio Imager Z1; Carl Zeiss, Oberkochen, Germany) integrated with a 10× or 20× objective lens and a computer-operated motorized stage; WU, FITC, and Texas Red filter sets; a digital camera (AxioCam HRc; Carl Zeiss); and AxioVision acquisition software (Carl Zeiss). Subsequently, image analysis had been performed and objects that satisfied predetermined criteria had been counted. Fluorescent intensities and morphometric characteristics, such as cell size, shape, and nuclear size, were considered when performing CTC identification and non-tumor cell exclusion, with cells characterized by a round to oval morphology and a visible nucleus (i.e., as Hoechst-33342 positive) that were positive for cytokeratin and negative for CD45 identified as CTCs. Isolated CTCs on the transparent MCA were also stained using a May-Grünwald–Giemsa (MGG) staining method consisting of fixation with 4% PFA, undiluted May-Grünwald stain for 2 min, May-Grünwald stain diluted 50% in PBS for 1 min, and Giemsa stain for 18 min, followed by rinsing with PBS for 1 min.

### CTC Enumeration using the CellSearch System

Whole blood samples were maintained at room temperature, mailed overnight to the laboratory of SRL Inc., and processed within 96 h of collection. All CTC evaluations were performed without knowledge of patient clinical status in the laboratory and the results were reported quantitatively as the number of CTCs/7.5 mL of blood. CTCs were defined as EpCAM-isolated intact cells showing positive staining for cytokeratin and negative staining for CD45. In accordance with previous evaluations of the CellSearch system [8], a patient was considered CTC positive if ≥2 CTCs/7.5 mL of blood were detected in the patient’s sample.

## Results

### CTC Isolation and Image Analysis using the MCA System

Isolation and staining of the tumor cells from whole blood was completed within 120–180 min, and image scanning of the MCA was performed at 3 fluorescence wavelengths using a 10× or 20× objective lens and a motorized stage. Figure 1b–f shows the scanning electroscope microscopy (SEM) and fluorescence images of the stained cells that were recovered on the MCA. As can be observed, solitary cells and cell clusters were individually trapped and retained on the microcavities that could be easily enumerated. Recovered cells that had a round to oval morphology and a visible nucleus (i.e., were Hoechst 33342 positive) and were positive for pancytokeratin and negative for CD45 were identified as tumor cells, while CD45-positive cells were identified as contaminating normal hematologic cells. The images reveal the existence of a distinct immunophenotype of epithelial cell marker-positive tumor cells. Although a number of leukocytes were retained on the array, tumor-cell enumeration was relatively facile because individual cells had been trapped on the precisely aligned microcavities.

### Sensitivity of the MCA System in CTC Detection of Lung Cancer Cell Lines

In our previous study, varying numbers of cells of the lung cancer cell line NCI-H358 were spiked into blood, and tumor cell isolation was evaluated using our MCA system [31]. The calculated detection efficiency was constant and over 90% when 10–100 tumor cells were present per milliliter of blood. In this study, in order to evaluate the recovery efficiency of various lung cancer cell lines using the MCA system, 100 cells of each of 8 lung cancer cell lines (A549, HCC-827, NCI-H358, NCI-H441, PC-14, DMS-72, NCI-H69, and NCI-H82) were spiked into healthy donor blood samples and then processed by MCA assay. Table 1 shows the average recovery efficiency and typical diameter of the cell lines. As can be observed, a high recovery rate was obtained, regardless of tumor type, ranging from 68% to 100% in the cell line spike-in experiments. Most of the recovered cells were viable and able to proliferate even after undergoing the isolation process, suggesting the potential for further biological and molecular analysis of CTCs.

**Table 1:** CTC recovery efficiency and average cell diameter.

Cell line	Origin	Average cell diameter (µm)	Recovery efficiency (%)
A549	NSCLC	17.3	98±3
HCC827	NSCLC	19.6	99±6
NCI-H358	NSCLC	18.1	100±6
NCI-H441	NSCLC	20.6	98±8
PC-14	NSCLC	19.5	97±2
DMS79	SCLC	14.1	76±1
NCI-H69	SCLC	12.5	68±2
NCI-H82	SCLC	13.5	80±4

Cells were spiked into 1 mL of normal blood and recovered using the MCA system.

**Figure 2:**
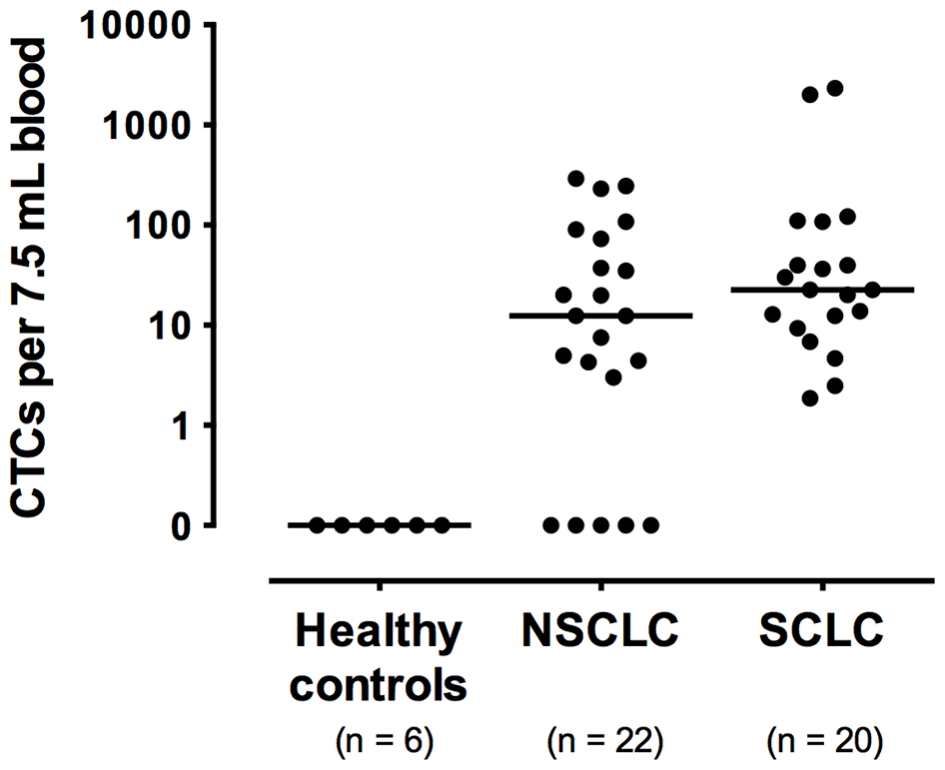
CTC count using the MCA system. CTC count/7.5 mL blood is shown for 6 healthy donors, 22 NSCLC patients and 20 SCLC patients.

Next, in order to evaluate the specificity and sensitivity of CTCs detection, the sensitivity tests were performed on artificial samples prepared by adding 1 and 3 cultured NCI-H358 cells to healthy donor blood samples, as previously reported by Vona et al. [20]. One and 3 cultured NCI-H358 cells were spiked into separate 7.5 mL aliquots of blood. These 7.5 mL blood samples were processed with the MCA system in 3 independent tests (Table S1). The results demonstrated a sensitivity threshold for MCA system close to 1 tumor cell per 7.5 mL of blood. In addition, CTCs were not detectable from 6 healthy donor bloods using the MCA system (Figure 2). Therefore, a patient was considered CTC positive if ≥1 CTCs per 7.5 mL of blood was detected by the MCA system.

In addition, the tumor cell recovery efficiency of the MCA system was compared with that of ISET system (Figure S1). In this comparison, 100 cells of NSCLC cell line NCI-H358 was spiked into healthy donor blood samples and then processed by the MCA system and a track-etched polycarbonate 8-µm pore membrane (Nucleopore; Whatman Ltd., Kent, UK). The results revealed the recovery rate using the MCA system (100% ±5%) to be significantly higher than that using the ISET system (91% ±2%) (p<0.05, t-test), indicating that use of the MCA system enables CTC isolation with an efficiency equivalent to or greater than that of the ISET system.

### CTC Enumeration using the CellSearch System and the MCA System

To conduct blind comparison of the detection sensitivity of the CellSearch and MCA systems, blood samples were collected from 22 metastatic NSCLC and 21 SCLC patients between April 2011 and February 2012 and analyzed for determination of the number of patients identified as CTC positive by each system (Table 2). Of these samples, 1 sample collected from 1 SCLC patient was not evaluated by the MCA system because an insufficient volume of blood had been collected for processing by both systems. As a result, 17 of the 22 (77%) NSCLC patients were identified as CTC positive using the MCA system but only 7 of the 22 (32%) NSCLC patients using the CellSearch system (Table 3). Of these patients, 8 were identified as CTC positive by both the CellSearch system and the MCA system, 1 was identified as CTC positive by the CellSearch system only, and 9 were identified as CTC positive by the MCA system only. Considering the results obtained by both systems together, 18 (82%) of the NSCLC patients were identified as CTC positive. Analysis of these findings revealed that a significantly greater number of NSCLC patients were identified as CTC positive by the MCA system (median cell count 13, range 0–291 cells/7.5 mL; Figure 2) than by the CellSearch system (median cell count 0, range 0–37 cells/7.5 mL), demonstrating the statistical superiority of the MCA system in CTC enumeration (p = 0.0015, Wilcoxon test; Table 3).

**Table 2:** Patient characteristics.

		NSCLC	SCLC
No. of patients		22	21
Gender	Male	10	18
	Female	12	3
Median age		68	73
	(Range)	(36–77)	(53–83)
Smoking	Smoker	16	21
	Never-smoker	6	-
ECOG-PS	0–1	16	13
	2–4	6	8
No. of organs with metastasis	Median	2	2
	(Range)	(1–6)	(1–5)
Metastasis	Brain	9	10
	Bone	8	4
	Liver	6	6
Histology	Adenocarcinoma	14	–
	Squamous	3	–
	Others	5	–
	SCLC	–	21

**Table 3:** Comparison of CTC enumeration by the CellSearch system and the MCA system.

Sample ID		CellSearch CTC (cells/7.5 mL)	MCA CTC (cells/7.5 mL)
**NSCLC**	1	0	0
	2	0	0
	3	9	0
	4	0	0
	5	0	5
	6	0	8
	7	2	90
	8	0	13
	9	0	13
	10	0	3
	11	1	35
	12	37	20
	13	2	246
	14	18	108
	15	0	73
	16	10	231
	17	19	20
	18	1	4
	19	0	0
	20	0	4
	21	0	291
	22	0	38
**SCLC**	23	200	20
	24	189	30
	25	0	13
	26	0	9
	27	0	40
	28	0	7
	29	33	23
	30	2	14
	31	3	122
	32	18	2
	33	1	2329
	34	1	2021
	35	4	13
	36	15	5
	37	325	40
	38	2	–
	39	13	36
	40	110	110
	41	0	3
	42	0	23
	43	0	109

In contrast, 20 of the 20 (100%) SCLC patients were identified as CTC positive using the MCA system versus 12 of the 21 (57%) patients using the CellSearch system. The median CTC count was found to be 2 cells/7.5 mL (range 0–325) using the CellSearch system and 23 cells/7.5 mL (range 2–2329) using the MCA system (Figure 2). Although not reaching a level of statistical significance, the detection sensitivity of the MCA system in CTC enumeration showed a trend toward being greater than that of the CellSearch system (p = 0.2888, Wilcoxon test; Table 3). For each outcome, agreement between the test results of the systems was assessed by Bland–Altman plots [33]. In the analysis of agreement regarding CTC enumeration in NSCLC patients, the mean difference was 50.1 (95% CI, range 11.1–89.1), with the limits of agreement ranging from −125.8 to 226.0. The MCA system yielded disproportionally higher CTC counts at higher mean values compared to The CellSearch system (Figure S2a). In contrast, in the analysis of agreement regarding CTC enumeration in SCLC patients, the mean difference was 202.6 (95% CI, range −116.7–521.9), with the limits of agreement ranging from −1162.0 to 1567.2. Unlike with the analysis of NSCLC blood samples, no bias was observed between the systems in the analysis of SCLC samples except for subjects with extremely high CTC titer (Figure S2b). Statistical analysis also revealed no association between site of metastasis and the CTC count of lung cancer patients using either system (data not shown).

### Morphologic Features of CTCs Isolated using the MCA System

**Figure 3:**
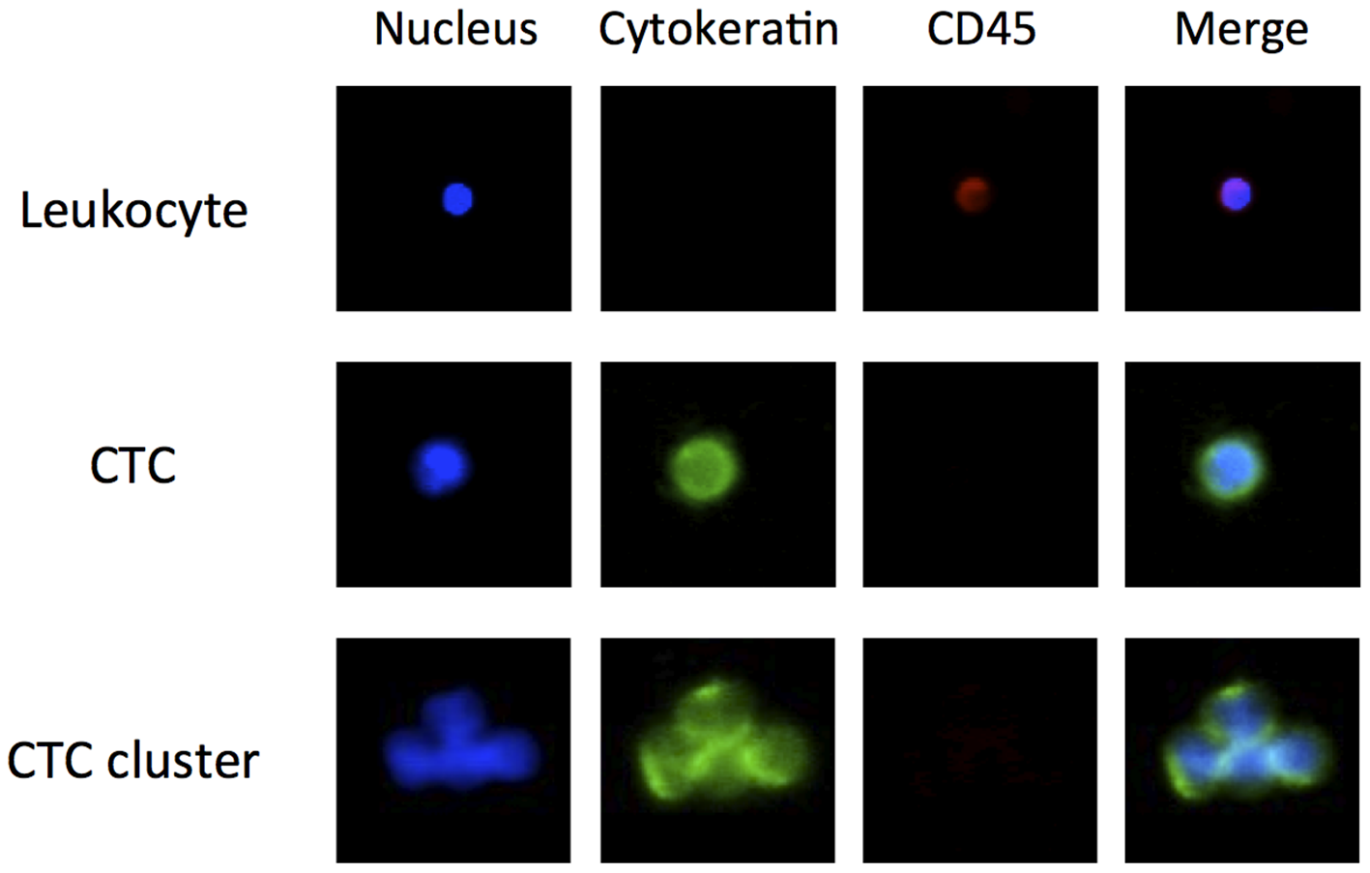
Gallery of cells captured on the MCA from blood of advanced lung cancer patients. Cells were stained with Hoechst 33342, FITC-labeled anti-cytokeratin antibody, and PE-labeled anti-CD45 antibody.

**Figure 4:**
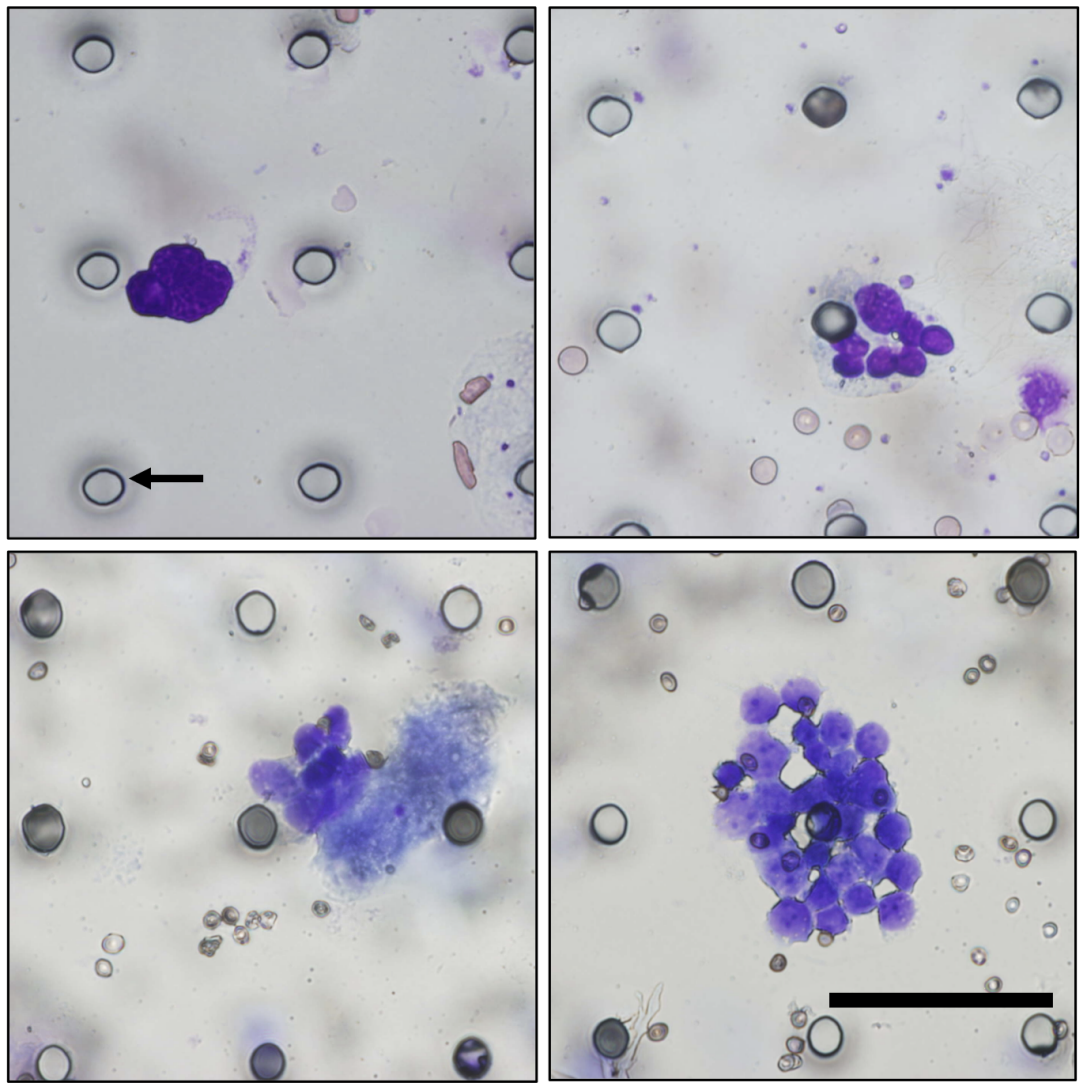
Gallery of CTCs captured on a transparent MCA from SCLC patient blood. May-Grünwald–Giemsa-stained cells showed a high nucleus–cytoplasm ratio and nuclear molding (×40). Black arrow indicates 9-µm microcavity. Scale bar = 60 µm.

CTCs were counted, identified as being cytokeratin positive and CD45 negative, and as having a visible nucleus on the basis of analysis of fluorescent images. As can be observed in Figure 3, which shows a representative gallery of CTCs identified by image analysis, CTCs are larger than the surrounding leukocytes and often appear in clusters, defined here as contiguous groupings of cells containing 3 or more nuclei. Figure 4 shows a solitary CTC and a CTC cluster detected in one SCLC patient using the MCA system. Using the MCA system, CTC clusters were observed in 2 of the 22 NSCLC patients (Patient No. 13 and 21) and 4 of the 21 SCLC patients (Patient No. 31, 33, 34, and 43). May-Grünwald–Giemsa staining of the CTCs isolated using the MCA system revealed that they are characterized by a high N/C ratio, nuclear molding, and morphological similarity to primary tumor cells.

## Discussion

ISET systems have been found to have higher CTC detection sensitivity than the CellSearch system in several cancers, including NSCLC [17], [22] However, the pores of ISET filters, which are made of polycarbonate by track etching, are randomly placed within the systems at a nonuniform density. Unlike such track-etched polycarbonate filters, the size, geometry, and density of the microcavities in the MCA system assessed in the current study are precisely controlled to achieve specific cell separation according to differences in cellular size and deformability. Aligning cells on the MCA not only eases cell imaging by allowing for the scanning of specified areas with an automated fluorescence microscope but also enables reduction in the labor required for CTC counting [29], [31]. As such, the MCA system provides a platform for the use of high-throughput imaging technologies that provide more rapid and less expensive data collection as well as CTC enumeration and advanced analysis of molecular phenomena, including fluorescence in situ hybridization for detection of tumor-specific genomic changes. Furthermore, the MCA is integrated with a miniaturized device so that enrichment of CTCs from blood, as well as staining and washing in the microfluidic assay, can be performed within one integrated device.

In the present study, CTCs isolated on the MCA were successfully stained with fluorescent-labeled antibodies that target tumor cell markers, and staining and washing were found to have little or no effect on the retention of tumor cells on the microcavities. Due to its very small size, the MCA system is portable, which, by enabling point-of-care CTC counting, eliminates the need to ship blood for testing under unfavorable shipment conditions and expedites clinical decision-making. These features, in addition to our recently developed procedure for isolating single cells from the MCA using microcapillaries, allow tumor cells to be recovered from the MCA for subsequent molecular analysis of CTCs [29].

In this blind comparison of use of the MCA system to that of the conventional CellSearch System for CTC enumeration in lung cancer patients, the MCA system was found capable of isolating various lung cancer cell lines spiked within whole blood at high levels of efficiency. However, the MCA system performed isolation of SCLC cell lines slightly less efficiently compared to that of NSCLC cell lines, indicating that small (<8 µm in diameter) cells of the SCLC cell lines might pass through the microcavities during blood filtration. In a previous study [31], we found that breast (MCF-7 and Hs578T), gastric (AGS and SNU-1), and colon (SW620) tumor cells lines that include EpCAM-negative tumor cells could be successfully recovered using the MCA system with greater than 80% efficiency. However, we also found that the efficiency of recovery of small cells (average diameter 11.6 µm) of the tumor cell line SW620 to be slightly less than that of other cell lines, as we did of the SCLC cell lines examined in this study.

The MCA system assessed in the present study was found to possess a higher detection sensitivity than the CellSearch system in NSCLC CTC enumeration, suggesting the superiority of size- and deformability-based isolation techniques compared to immunomagnetic-based techniques. The poor sensitivity of CellSearch has been attributed to the low EpCAM expression in advanced NSCLC. However, one of the NSCLC patients assessed in the present study was found to be CTC positive using the CellSearch system but CTC negative using the MCA system, indicating that changes in EpCAM expression cannot solely account for the differences found between the two systems in NSCLC enumeration.

The CTC detection rate using the CellSearch system in SCLC patients was 67%, considerably higher than that in NSCLC patients and consistent with that found in previous studies [7], [34]–[36]. Although the MCA system does not rely on EpCAM expression, which circulating SCLC cells have been reported to show high levels [37], in performing CTC isolation, its use was found to yield a high detection rate, indicating that it could be utilized for CTC detection in not only NSCLC but also SCLC patients. Nevertheless, the CTC counts of several patients were higher when analyzed using the CellSearch System compared to the MCA system, indicating that some small tumor cells in patient blood might flow through the microcavities, as described above. Previous research has suggested that immunomagnetic separation techniques lack the capacity to isolate large clusters, whereas use of size-based separation techniques leads to loss of small CTCs [17]. To address these problems, the shape of the microcavities in the MCA was modified to improve their efficiency in isolating small cells from tumor cells in whole blood in our recent study [38].

Observation of CTC clusters has been reported in various cancers, including lung cancer [23], [24], [39]–[42]. It is hypothesized that forming in clusters provides CTC cells with advantages over remaining solitary in terms of survival, proliferative capacity, and ability to form micrometastases. In this study, CTC clusters were isolated from both NSCLC and SCLC patients using the MCA system. Interestingly, the CTC-positive clusters were identified as having a small number of CTC cells by the CellSearch system but a large number by the MCA system. One reason why several SCLC patients were found to have a large CTC count when assessed by the MCA system may be that this system enables isolation of larger CTC clusters that cannot be isolated by immunomagnetic separation. Examination of this hypothesis requires further detailed analysis of the characteristics of CTC clusters, such as expression of epithelial markers and the presence of apoptotic cells within CTC clusters, which could be performed using the MCA system.

In conclusion, our results suggest that the MCA system is potentially superior to the CellSearch system in the CTC detection of lung cancer patients, with the former found capable of isolating significantly more CTCs and CTC clusters than the latter. The major limitation of this study was its examination of a small sample of patients with only one type of cancer. Further studies should thus examine larger cohorts of patients with various types of cancers to assess whether the MCA system is a more appropriate tool for CTC enumeration and characterization of metastatic tumors in patients with cancers other than lung cancer compared to other systems. We are currently planning the development of an automated MCA system that achieves robust, reliable, and reproducible sample processing for validation study using large cohorts of patients presenting at multiple institutes to assess the prognostic utility of CTC count in cancer patients.

## Supporting Information

Figure S1 **Comparison of cell recovery rate using the microcavity array (MCA) system and an isolation by size of epithelial tumor cell (ISET) filter.** Non-small cell lung cancer cell line NCI-H358 was spiked into whole blood at a volume of 100 cells/mL to perform 3 separate tests of circulating cancer cell recovery using an MCA (pore size = 8 µm) and a track-etched polycarbonate ISET filter (pore size = 8 µm; Nucleopore).
(TIFF)

Figure S2 **Bland–Altman plots of agreement between circulating tumor cell (CTC) test results obtained for non-small cell lung cancer (NSCLC; a) and small cell lung cancer (SCLC; b) patients using the CellSearch and microcavity array (MCA) systems.** The solid horizontal line represents the mean difference and the dashed lines the limits of agreement (mean difference +/−2SD). In NSCLC, the mean difference was 50.1 (95%CI, 11.1 to 89.1), limits of agreement (-125.8 to 226.0) with the difference between systems becoming disproportionately greater with higher average CTC-count. In SCLC, the mean difference was 202.6 (95%CI, −116.7 to 521.9), limits of agreement (-1162.0 to 1567.2) with no bias observed between systems except for subjects with extremely high titer of CTCs.
(TIFF)

Table S1 Evaluation of sensitivity of microcavity array (MCA) system for circulating tumor cell (CTC) detection. Sensitivity testing was performed using artificial samples created by adding 1 and 3 cultured NCI-H358 cells to healthy donor blood samples. Individual cells were selected by micropipette under direct visualization, spiked into 7.5 mL aliquots of blood, and the resulting blood samples processed using the MCA system in 3 separate tests.
(DOC)
