# Supplementary figures and images for: Size-Based Isolation of Circulating Tumor Cells in Lung Cancer Patients Using a Microcavity Array System
Source: PLoS One. 2013 Jun 28;8(6):e67466. doi: 10.1371/journal.pone.0067466 (PMC3696066; doi:10.1371/journal.pone.0067466)

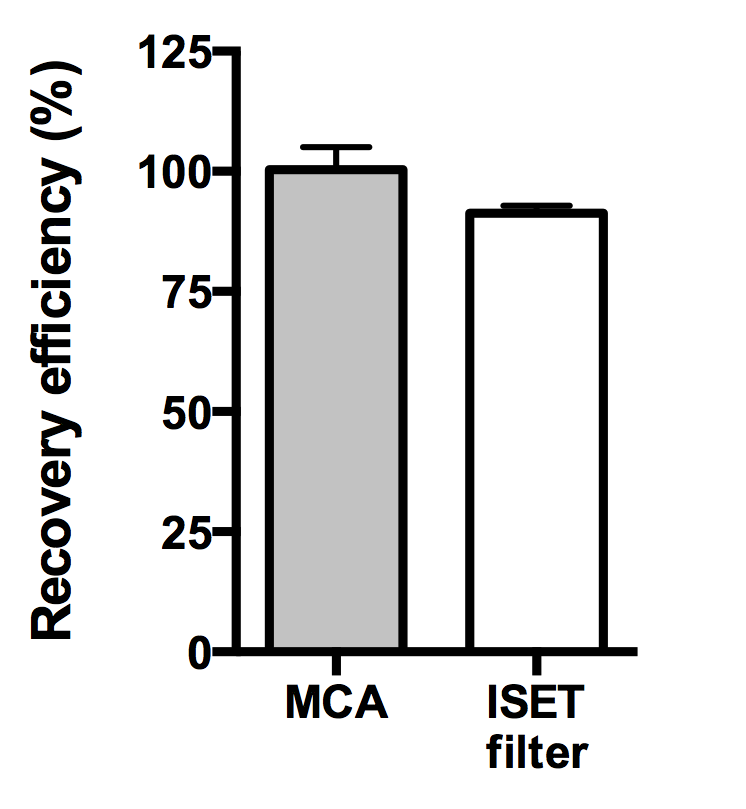

Supplement: Figure S1 — Comparison of cell recovery rate using the microcavity array (MCA) system and an isolation by size of epithelial tumor cell (ISET) filter. Non-small cell lung cancer cell line NCI-H358 was spiked into whole blood at a volume of 100 cells/mL to perform 3 separate tests of circulating cancer cell recovery using an MCA (pore size = 8 µm) and a track-etched polycarbonate ISET filter (pore size = 8 µm; Nucleopore). (TIFF) [file pone.0067466.s001.tif]

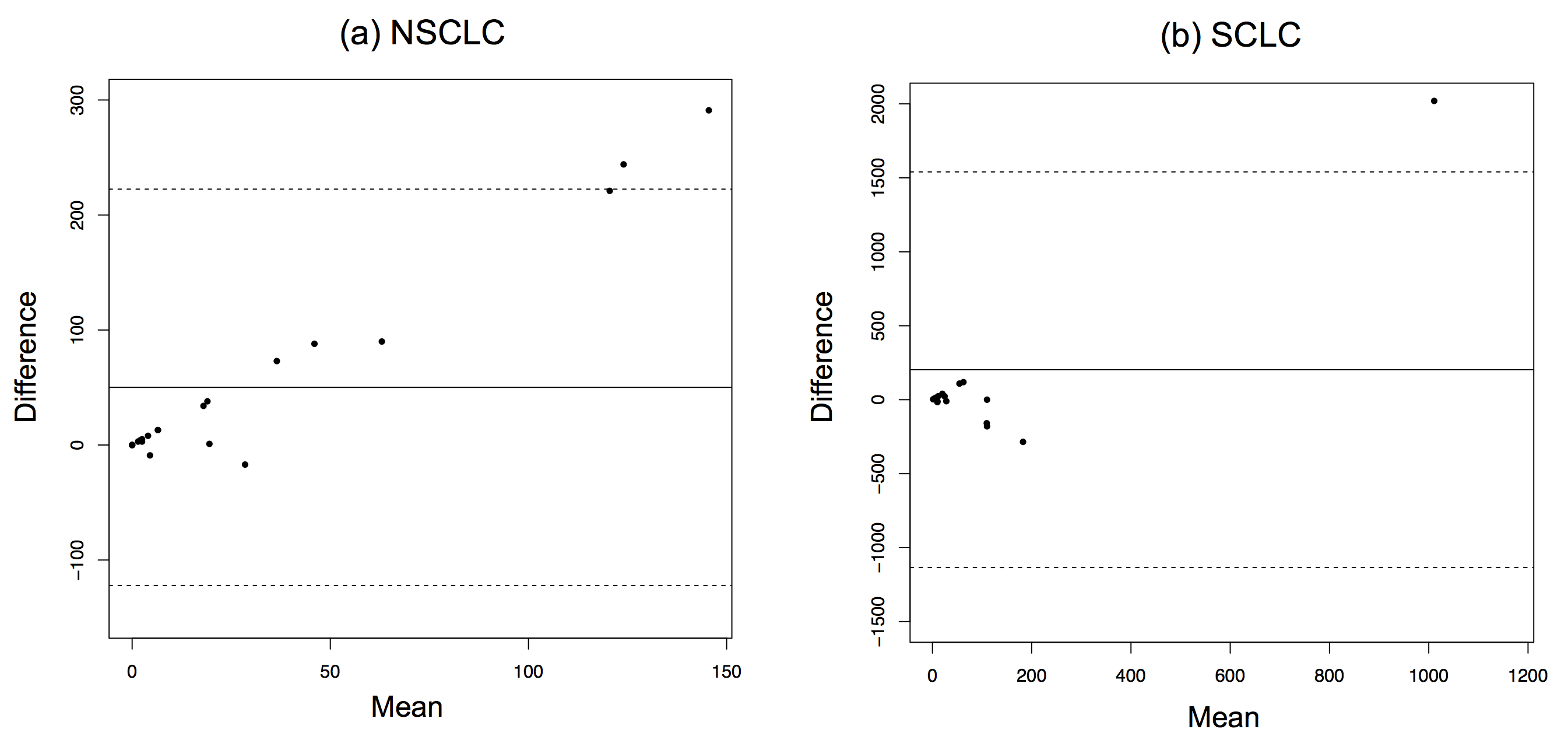

Supplement: Figure S2 — Bland–Altman plots of agreement between circulating tumor cell (CTC) test results obtained for non-small cell lung cancer (NSCLC; a) and small cell lung cancer (SCLC; b) patients using the CellSearch and microcavity array (MCA) systems. The solid horizontal line represents the mean difference and the dashed lines the limits of agreement (mean difference +/−2SD). In NSCLC, the mean difference was 50.1 (95%CI, 11.1 to 89.1), limits of agreement (-125.8 to 226.0) with the difference between systems becoming disproportionately greater with higher average CTC-count. In SCLC, the mean difference was 202.6 (95%CI, −116.7 to 521.9), limits of agreement (-1162.0 to 1567.2) with no bias observed between systems except for subjects with extremely high titer of CTCs. (TIFF) [file pone.0067466.s002.tif]
