# Supplementary material for: Size-Based Isolation of Circulating Tumor Cells in Lung Cancer Patients Using a Microcavity Array System
Source: PLoS One. 2013 Jun 28;8(6):e67466. doi: 10.1371/journal.pone.0067466 (PMC3696066; doi:10.1371/journal.pone.0067466)
Supplement: Table S1 — Evaluation of sensitivity of microcavity array (MCA) system for circulating tumor cell (CTC) detection. Sensitivity testing was performed using artificial samples created by adding 1 and 3 cultured NCI-H358 cells to healthy donor blood samples. Individual cells were selected by micropipette under direct visualization, spiked into 7.5 mL aliquots of blood, and the resulting blood samples processed using the MCA system in 3 separate tests. (DOC) [file pone.0067466.s003.doc]

**Table S1. Evaluation of sensitivity of microcavity array (MCA) system for circulating tumor cell (CTC) detection.**

Sensitivity testing was performed using artificial samples created by adding 1 and 3 cultured NCI-H358 cells to healthy donor blood samples. Individual cells were selected by micropipette under direct visualization, spiked into 7.5 mL aliquots of blood, and the resulting blood samples processed using the MCA system in 3 separate tests.

|  | No. of cells detected | | | Cell recovery efficiency (%) | |
| --- | --- | --- | --- | --- | --- |
| No. of cells spiked | Test 1 | Test 2 | Test 3 | Average | SD |
| 1 | 0 | 1 | 1 | 67 | 58 |
| 3 | 2 | 2 | 3 | 78 | 19 |
